# Supplementary figures and images for: Unlocking the Potential of Camel Milk-Derived Exosomes as Novel Delivery Systems: Enhanced Bioavailability of ARV-825 PROTAC for Cancer Therapy
Source: Pharmaceutics. 2024 Aug 15;16(8):1070. doi: 10.3390/pharmaceutics16081070 (PMC11359469; doi:10.3390/pharmaceutics16081070)

CD63

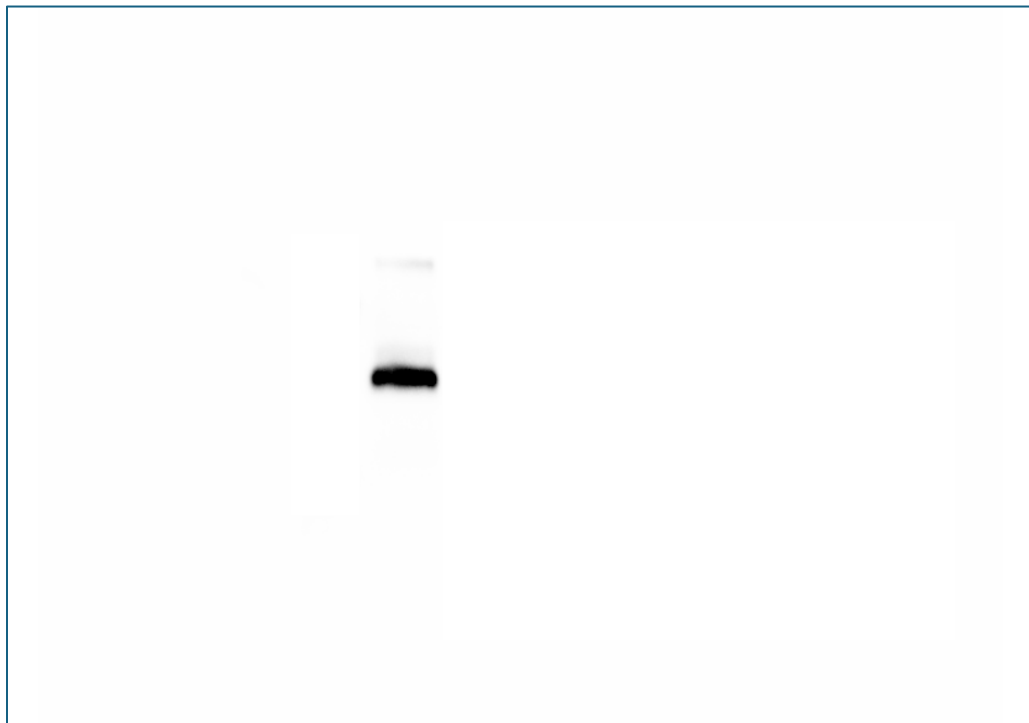

CD81

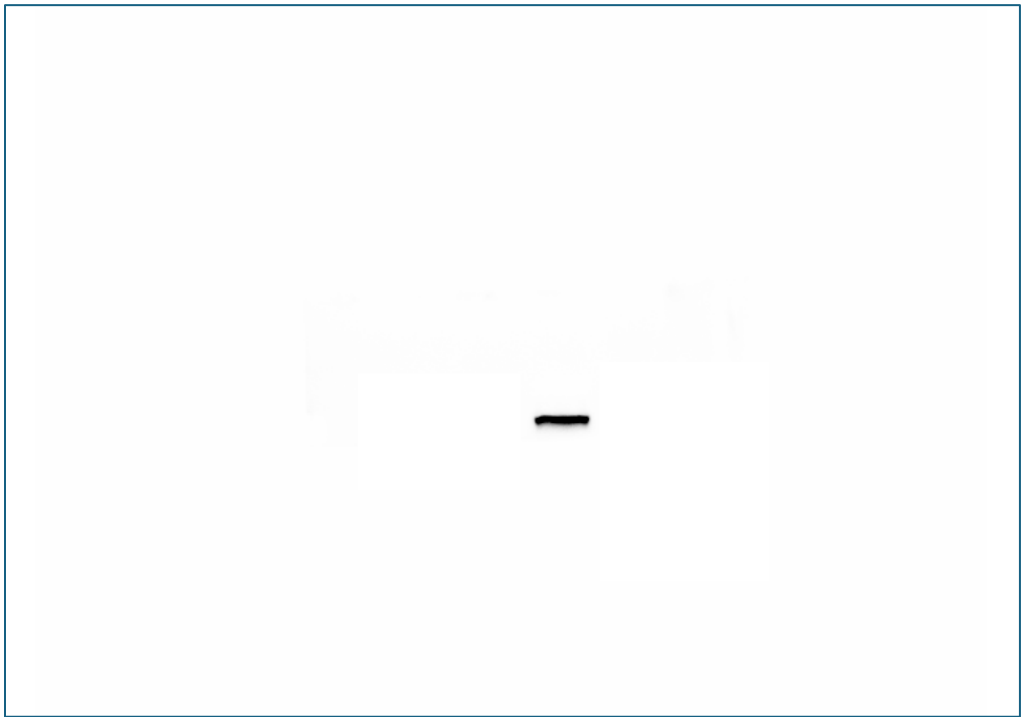

ALIX

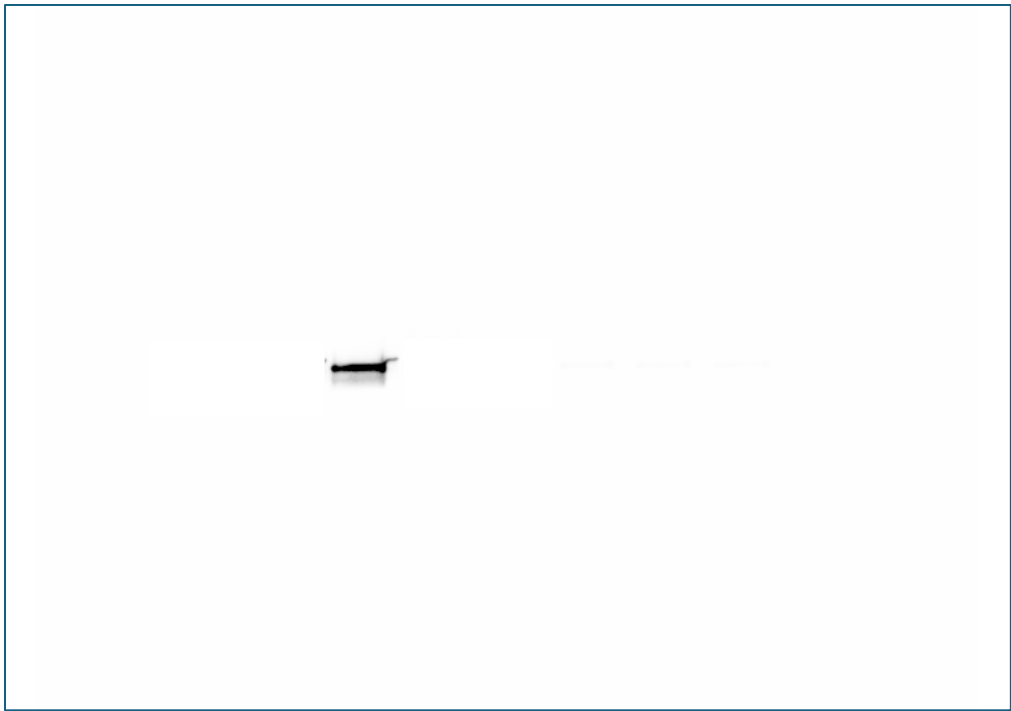

HSP70

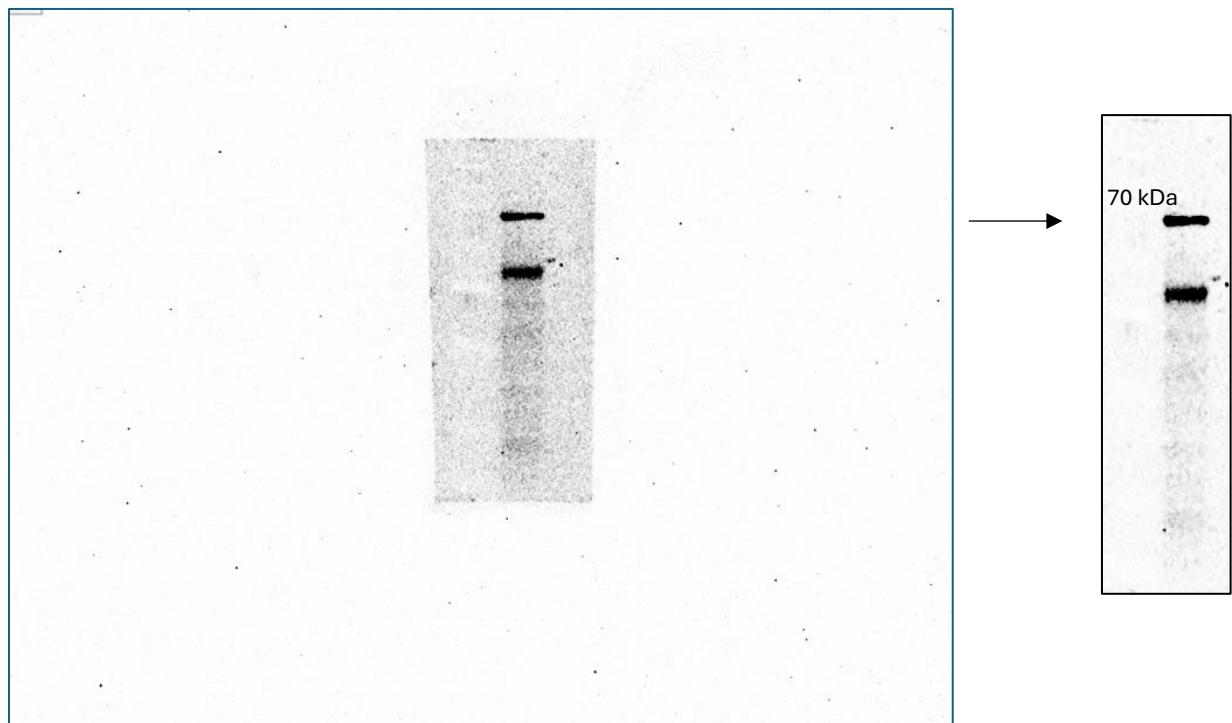

Supplement: Supplementary file 1 [file pharmaceutics-16-01070-s001.zip › Western blots.pdf]
